# Screening policies, preventive measures and in-hospital infection of COVID-19 in global surgical practices

**DOI:** 10.7189/jogh.10.020507

**Published:** 2020-12

**Authors:** Vittoria Bellato, Tsuyoshi Konishi, Gianluca Pellino, Yongbo An, Alfonso Piciocchi, Bruno Sensi, Leandro Siragusa, Krishn Khanna, Brunella Maria Pirozzi, Marzia Franceschilli, Michela Campanelli, Sergey Efetov, Giuseppe S Sica, on behalf of S-COVID Collaborative Group, A Kefleyesus, AGM Hoofwijk, Abdullah Sami Eldaly, Abel Gonzalez, Adraoui Jawad, Ahmad Jooma, Ahmed Mousa Hafez, Ainhoa Valle Rubio, Aitor Landaluce-Olavarria, Aiwen Wu, Akihisa Nagatsu, Akira Inoue, Akira Kanamoto, Akira Ouchi, Alaa El-Hussuna, Alba Vazquez-Melero, Albert M Wolthuis, Alberto M Peral, Alejandra Cruz Lozano, Aleksandr Efremov, Aleksandr V Ryasantsev, Alessandra Di Giorgio, Alessandro Parente, Alessandro Tamburrini, Alessio Alò, Alexander Forero-Torres, Alexander L Vahrmeijer, Alexander Varabei, Siles Hinojosa, Ali Zeynel Abidin Balkan, Alice Frontali, Alikin Oleg, Álvaro Soler-Silva, Amin Makni, Ana André, Ana María García Cabrera, Ana María Gonzalez Fernández, Ana M Minaya-Bravo, Ana Rodríguez-Sánchez, Ana-Maria Musina, Anang Pangeni, Anastasia Zolotko, Andranik Tonoyan, Andrea Balla, Andrea Belli, Andrea Cavallaro, Andrea Chierici, Andrea Divizia, Andrea Fares Bucci, Andrea Jiménez Salido, Andrea Morini, Andrea Muratore, Andrea Vignali, Andrei Chitul, Diaconescu Andrei Sebastian, Andrejs Pcolkins, Andrey Shchegolev, Andrew Hollenbeck, Andrew Wisneski, Angelo Iossa, Anna D’Amore, Anndrew Hunter, Anthony J Hesketh, Andrea La Brocca, Antonino Spinelli, Antonio Caires, Antonio D’Alessandro, Antonio Francisco Sanchís López Correo, Antonio Macrì, Antonio Navarro-Sánchez, Apollo Pronk, Aram Akunc, Arash Mehri, Arie Pelta, Aristeidis Papadopoulos, Aristotelis Kechagias, Arshad Rashid, Artur Ramazanov, Ashfaq Chandio, Atsushi Kohyama, Atsushi Nishimura, Atsushi Ohkawa, Audrius Dulskas, Aun Jamal, Aurora Mariani, Ayse Gizem Unal, Ayse Karagoz, Bahar Busra Ozkan, Barham Salih, Baris Gülcü, Beatrice Pessia, Beatriz Martin-Perez, Benedetto Ielpo, Berenice Tulelli, Bin Yang, Boumadani Mhamed, Brenda Murphy, Bs Langenhoff, Bulent Belevi, Burak Güney, Caecilia Ng, Camilo Rueda, Campbell S Roxburgh, Carlo V Feo, Carlo Ferrari, Carlo Gazia, Carlo Pratesi, Carlo Ratto, Carlos Cerdán Santacruz, Carlos Rodolfo Martinez Arroyave, Carlos Macias, Carlota Garcia Fernandez, Carmen Cagigas Fernandez, Carolina Curtis-Martinez, Caroline Fortmann, Caroline Kim, Catalina Uribe Galeano, Catarina Barroso, Caterina Baldi, Caterina Foppa, Cesare Formisano, Changzai Li, Chao Ding, Chenyu Wang, Chiara Iacusso, Chongwei Yang, Christian Pizzera, Christoph Skias, Christos Chouliras, Christos Liakos, Chu Matsuda, Chun-yi Wu, Cihangir Ozaslan, Cinzia Tanda, Cipolat Mis Tommaso, Claire Dagorno, Claudia Patricia Arellano Ramos, Claudio Arcudi, Claudio Coco, Cleotilde Mateo Morales, Mujahid Zulfiqar Ali, Constança Teresa Miranda De Azevedo, Coral Cózar Lozano, Corrado Sala, Cosimo Alex Leo, Cosimo Riccardo Scarpa, Cristian Varela Ferro, Cristina Mosquera Fernandez, D Morales-Garcia, Daisuke Nakano, Daniel Cristian, Daniel Hechtl, Daniel Triguero Cánovas, Daniela Calabrese, Daniela Rega, Daniele Ferraro, Daniele Morezzi, Daniele Sommacale, Danielle Brogden, Danilo Miskovic, David Merlini, Davide Pertile, Denise Coniglio, Dexiang Zhu, Dianwen Wu, Diego Coletta, Diego Ramos Rubio, Diego Sasia, Dmitry Fillipov, Domenico Russiello, Dragomir Dardanov, ECJ Consten, Edgaras Smolskas, Edoardo Maria Muttillo, Edward Jones, Eiji Sunami, El-Helou Etienne, Elena Chalkiadaki, Elena Giacomelli, Elena Karbovnichaya, Elena Ruiz-Úcar, Eleonora Guaitoli, Elgun Samadov, Elio Jovine, Elio Treppiedi, Elisa Maria Vaterlini, Elisa Zambaiti, Elisabetta Moggia, Elmi Coetzee, Emanuele Chisari, Emanuele D’Errico, Emilia Ciofic, Emilio Peña, Emine Kurt, Emre Balık, Emre Gunay, Emre Sivrikoz, Enrico Andolfi, Enrico Araimo, Enrico Lucci, Enrico Opocher, Enrico Pinotti, Enrico Rubino, Enver Reyhan, Erica Mazzotta, Ernesto Barzola Navarro, Etienne El-Helou, Eugenio Licardie-Bolaños, Eva Iglesias Porto, Evelyn Contreras, Evert-Jan Boerma, Fabio Cianchi, Fabio Marino, Fabio Uggeri, Fanghai Han, Federica Calculli, Federica Falaschi, Federico Ghignone, Federico Perrone, Felice Borghi, Felipe García, Ferdinando Agresta, Ferdinando Carlo Maria Cananzi, Fernando Mendoza-Moreno, Fevzi Cengiz, Filipe Macedo Almeida, Filippo Baracchi, Filippo Carannante, Filippo La Torre, Flavio Fernandes, Florian Friedmacher, Florin Grama, Francesca Carissimi, Francesca Pecchini, Francesco Bianco, Francesco Colombo, Francesco Ferrara, Francesco Litta, Francesco Maria Carrano, Francesco Martignoni, Francesco Menegon Tasselli, Francesco Milone, Francesco Pata, Francesco Sammartino, Francesco Zambianchi, Francisco Barragan, Francisco Herrero, Francisco Schlottmann, Frank C Den Boer, Frank Pfeffer, Fumihiko Fujita, G Navarra, Gabriel Herrera-Almario, Gabriele Pozzo, Gabriella Teresa Capolupo, Gabrielle H Van Ramshorst, Gadiel Liscia, Gaetano Gallo, Ganesh Asawa, Gaoxiang Wang, Gaurang Raiyani, Geerard Beets, Gemma Sugrañes Naval, Genfeng Jin, George J Chang, George Saakian, Gerardo Kahane, Giacomo Borroni, Giacomo Lo Secco, Gian Luca Baiocchi, Gianluca Baronio, Gianluca Pagano, Gianmaria Casoni Pattacini, Giorgio Lisi, Giovanni Milito, Giovanni Sinibaldi, Giuditta Serrao, Giulia Bagaglini, Giuliano Sarro, Giuseppe Brisinda, Giuseppe Candilio, Giuseppe Mangiameli, Giuseppe Giuliani, Gonzalo Pablo Martin-Martin, Gordon Bodzin, Graat Leon, Graham Mackay, Granila Vasil, Graziano Palmisano, Grella Maria Giovanna, Guadalupe Campos Fernández, Guillermo Berrones Steingel, Guoyun Zhang, Gyu Seog Choi, Haipeng Chen, Hajime Hirose, Hajime Kayano, Hanife Seyda Ulgur, Harmony Impellizzeri, Hasani Ariola, Heli Liu, Heriberto Medina, Hideaki Miyauchi, Hidekazu Takahashi, Hideki Hayashi, Hideki Ishikawa, Hideyuki Ishida, Hilbert De Vries, Hilmican Ulman, Hirofumi Kon, Hirofumi Ota, Hiroki Akamatsu, Hiroshi Tamagawa, Hirotaka Shoji, Hiroyuki Egi, Hisahiro Matsubara, Hisanori Miki, Hossam Elfeki, Hung-Hsin Lin, Iacopo Giani, Iban Caravaca-García, Ichiro Takemasa, Imerio Angriman, Ionut Negoi, Irina Volkova, Iris Russo, Irmgard E Kronberger, Iskander Shageev, Ishak Aydin, Ismael Mora-Guzmán, Ivana Novak, Izzo Giuliano, Jacob Rachmuth, James Chi-Yong Ngu, James Glasbey, Jan Stoot, Jan Žatecký, Jarno Melenhorst, Van Der Wal JBC, Jeroen Leijtens, Jessica Bogach, Jessie Elliott, De Wilt JHW, Jiagang Han, Jian Cui, Jiaqi Liu, Jim Khan, Jimmy Panji Wirawan, Jinji Zhang, Joel Davis Osorio Manyari, Johannes Doerner, Jonathan Bock, Joop Konsten, Jorge Mario Castro, Jorge Pérez Grobas, José Pereira Pinto, Jovan Juloski, Juan Luis Blas Laina, Juan José Solórzano, Juan Ramón Gómez López, Jun Li, Jun Watanabe, Jung-Myun Kwak, Junichi Hasegawa, Junichiro Hiro, K Sergey, Kai Zhang, Kaoru Nagahori, Karla Martinez, Katsuji Tokuhara, Katsuki Danno, Kay Uehara, Kazuhiko Yoshimatsu, Kazuhisa Ehara, Kazuki Ueda, Kazuyoshi Suda, Kazuyuki Yamamoto, Kei Ishimaru, Kei Kimura, Keiji Hirata, Kemal Deen, Ken Imaizumi, Jenji Yamada, Kenta Tanakura, Khaled Rida, Kiichi Sugimoto, Kitani Kotaro, Kiwisure Shi, Koji Okabayashi, Koya Hida, Kozo Kataoka, Kumiko Hongo, Kunkun Xia, Larissa Tseng, Lars Reime, Laura Lorenzon, Laura Muiños Ruano, Lei Zhou, Lindsey De Nes, Lorena Brandariz, Lorenzo Morini, Lorenzo Petagna, Lorenzo Ripamonti, Lourdes Hernandez Martinez, Luca Pio, Luca Sacco, Lucia Carvalho, Luigi Zorcolo, Luis Eduardo Pérez-Sánchez, Luis Humberto Reyes Esparza, Luis Tallon Aguilar, Madeleine Garner, Maki Sugimoto, Makoto Nagashima, Manabu Shiozawa, Manfredelli Simone, Manuel Ferrer-Marquez, Marcia Carvalho, Marco Alifano, Marco Arganini, Marco Calussi, Marco Catarci, Marco Ettore Allaix, Marco Forlin, Marco Milone, Marco Paci, Margot Fodor, Maria Antipova, Maria Beltran Martos, Maria Carmela Giuffrida, María Diez Tabernilla, María José Alcaide Quirós, Maria Lemma, Maria Luisa Reyes Diaz Correo, Maria Małowiecka, Maria Paola Bellomo, Maria Ramos Fernandez, María Socias, Mariam Rizk, Mariani Aurora, Mariano Alvarez Antolinez, Marijana Ninkovic, Mario Giuffrida, Marjolein MN Leeuwenburgh, Marnix AJ De Roos, Marta Cañón Lara, Marta Climent Agustin, Marta Cuadrado, Marta Pascual, Martina Lemmerer, R Carlos, Masa Okamoto, Masaaki Miyo, Masafumi Inomata, Masakazu Ikenaga, Masaki Tsujie, Masamichi Yasuno, Masanori Kotake, Masanori Sato, Masayoshi Yasui, Matteo Lavazza, Matteo Rottoli, Matteo Zuin, Mauricio Zuluaga, Maurizio Cervellera, Maurizio Cesari, Maurizio Zizzo, Mauro Garino, Mauro Ghirardi, Mauro Montuori, Mauro Podda, Mauro Santarelli, Mehmet Ali Koc, Melissa Baini, Michael De Cillia, Michele De Rosa, Michele Manigrasso, Michele Zuolo, Miguel F Cunha, Mihaela Misca, Mihail Slavchev, Mikhail Danilov, Mikhail Shigaev, Milou Martens, Minako Kobayashi, Mingyang Ren, Mitsuru Ishizuka, Mohammed Mustafa Hassan, Mohamad Siblini, Mohamed Sahloul, Mohammad Reza Keramati, Monish Karunakaran, Moritz Markel, Mudassar Majeed, Muhammad Umar Younis, Muhammed Ikbal Akin, Munazza Laraibe, Murat Derebey, Murat Kendirci, Mutsumi Fukunaga, Nagahide Matsubara, Narce Eunice Cruz Ordaz, Narimantas Evaldas Samalavicius, Nattawut Keeratibharat, Nicola de Angelis, Nicolae Gica, Nicoló Maria Mariani, Nicolò Ramino, Nicolò Falco, Neil Smart, Niels De Korte, Niels FM Kok, Nigel B Jamieson, Nikolay Aberyasev, Nikolay Bruklich, Nobuki Ichikawa, Norikatsu Miyoshi, Norma De Palma, Nuno Figueiredo, Nuria Ortega Torrecilla, Oleg G Dybov, Oleg Yudin, Ollende Crepin, Oscar Gomez, Ozlem Zeliha Sert, Pablo Lozano Lominchar, Pablo Menéndez, Paola De Nardi, Patricia Tejedor, Patrick Jordan, Patrick Tan, Patrizia Marsanic, Pechnikova Natalya, Pedro Parra Baños, Pere Rebasa, Peter M Neary, Pieter Tanis, Piero Giustacchini, Pietro Anoldo, Pilar Concejo, Pin Cao, Pramodh Chandrasinghe, Prasad Abeyratne, Quan Wang, RJ Klicks, R Mukai, Rafael Ferrer Riquelme, Raffaele De Luca, Raffaele Galli, Raffaele Gianesini, Rajesh Gianchandani Moorjani, Rajkiran K Deshpande, Ramon Gorter, Raquel Leon Ledesma, Rategov Ruslan, Raunaq Chhabra, Reena Talreja-Pelaez, Rei Suzuki, Riccardo Balestri, Riccardo Rosati, Rim Kiblawi, Rita Martins, Roberta Angelico, Roberta Tutino, Roberto Persiani, Roberto Pollastri, Rocío González López, Rodrigo Oliva Perez, Roel Hompes, Roman Lukanin, Sera R Roser Termes, Rossella Brunaccino, Ryota Nakanishi, Samuel Stefan, Sandra Paola Sánchez Hernández, Sara Di Carlo, Sara Ingallinella, Satoru Domoto, Satoshi Ikeda, Saulius Mikalauskas, Seon Hahn Kim, Serena Mantova, Severius Barbuta, Shaotang Li, Shigeki Yamaguchi, Shigeru Yamagishi, Shigenori Homma, Shingo Tsujinaka, Shinichi Yoshioka, Shinichiro Mori, Shirish Tewari, Shlomi Rayman, Sho Horiuchi, Shuichiro Matoba, Shunji Morita, Sibel Yaman, Silvia Vigna, Silvio Testa, Simon Ng, Simona Deidda, Simone Cicconi, Simone Di Maria, Simone Sibio, Siyar Ersoz, Sofija Pejkova, Soliman Altarifi, Songbing He, Songphol Malakorn, Sosef Meindert, Sosuke Sumikawa, Stavan Parmar, Stefan Uranitsch, Stefano D’ugo, Stefano Giuliani, Stéphanie Breukink, Suk-Hwan Lee, Taishi Hata, Takahisa Ishikawa, Takashi Akiyoshi, Takashi Azuma, Takaya Kobatake, Takayuki Fukuzaki, Takeshi Aiyama, Takeshi Yamada, Tatiana Garmanova, Tatiana Gómez-Sánchez, Tatsuro Yamaguchi, Teresa De Jesús Flores, Teruyuki Usub, Tetsuhiro Tsuruma, Tetsuichiro Shimizu, Tihomir Georgiev Hristov, Ting Van Loon, Tohru Funakoshi, Tommaso Maria Manzia, Tomomichi Kiyomatsu, Tomonari Katayama, Tomonori Akagi, Tsunekazu Mizushima, Uemura Kazuhito, Ugo Elmore, Ugo Grossi, Vadim A Truchalev, Valentina Sosa Rodríguez, Valentina Testa, Valeria Tonini, Valerio Celentano, Valery M Nekoval, Vanessa Bettencourt, Vasif Mammadov, Verónica Alejandra Galue Leyva, Veronica Georgina Ortega Mariscal, Victor Edmond Seid, Victor Klemann, Víctor Turrado-Rodriguez, Vincenzo Papagni, Vincenzo Vento, Vincent Frering, Vincenzo Vigorita, Vitaliva V Petrove, Vladimir Lyadov, Wei Fu, Wei Mi, Woon Kyung Jeong, Wouter KG Leclercq, Xavier De Sousa, Xing Zhao, Xinxiang Li, Xinxin Wang, Xuanhua Yang, Xuelei Zhang, Ya'nan Zhen, Yan Dong, Yana Erushevich, Yasumasa Takii, Yasuo Sumi, Yeray Trujillo Loli, Yosef Lishtzinsky Yifat, Yoshifumi Shimada, Yoshihiro Nabeya, Yoshihito Ide, Yuan Wu, Yuichiro Tsukada, Yuji Miyamoto, Yuji Toiyama, Yujiro Fujie, Yuka Kaneko, Yukako Mokutani, Yuki Fujii, Yukihide Kanemitsu, Yulia Medkova, Yulong Chen, Yurema Gonzalez Ruiz, Yusuke Kinugasa, Zacaria Sow, Zeeshan Razzaq, Zejun Wang, Zheng Liu, Zhenguo Han, Zhihui Tai, Zhiyong Lai, Zi Qin Ng, Zilvinas Dambrauskas

**Affiliations:** 1 Department of Surgery, Minimally Invasive Unit, Università degli Studi di Roma “Tor Vergata”, Rome, Italy; 2 Department of Gastroenterological Surgery, Cancer Institute Hospital of the Japanese Foundation for Cancer Research, Tokyo, Japan; 3 Department of Advanced Medical and Surgical Sciences, Università degli Studi della Campania “Luigi Vanvitelli”, Naples, Italy; 4 Department of Colorectal Surgery, Vall d'Hebron University Hospital, Barcelona, Spain; 5 Department of General Surgery, Beijing Friendship Hospital, Capital Medical University, Beijing, China; 6 GIMEMA Foundation, Rome, Italy; 7 Department of Orthopaedic surgery, Rush University Medical Center, Chicago, IL, USA; 8 Department of Surgery, I.M. Sechenov First Moscow State Medical University, Moscow, Russia

## Abstract

**Background:**

In a surgical setting, COVID-19 patients may trigger in-hospital outbreaks and have worse postoperative outcomes. Despite these risks, there have been no consistent statements on surgical guidelines regarding the perioperative screening or management of COVID-19 patients, and we do not have objective global data that describe the current conditions surrounding this issue. This study aimed to clarify the current global surgical practice including COVID-19 screening, preventive measures and in-hospital infection under the COVID-19 pandemic, and to clarify the international gaps on infection control policies among countries worldwide.

**Methods:**

During April 2-8, 2020, a cross-sectional online survey on surgical practice was distributed to surgeons worldwide through international surgical societies, social media and personal contacts. Main outcome and measures included preventive measures and screening policies of COVID-19 in surgical practice and centers’ experiences of in-hospital COVID-19 infection. Data were analyzed by country’s cumulative deaths number by April 8, 2020 (high risk, >5000; intermediate risk, 100-5000; low risk, <100).

**Results:**

A total of 936 centers in 71 countries responded to the survey (high risk, 330 centers; intermediate risk, 242 centers; low risk, 364 centers). In the majority (71.9%) of the centers, local guidelines recommended preoperative testing based on symptoms or suspicious radiologic findings. Universal testing for every surgical patient was recommended in only 18.4% of the centers. In-hospital COVID-19 infection was reported from 31.5% of the centers, with higher rates in higher risk countries (high risk, 53.6%; intermediate risk, 26.4%; low risk, 14.8%; *P* < 0.001). Of the 295 centers that experienced in-hospital COVID-19 infection, 122 (41.4%) failed to trace it and 58 (19.7%) reported the infection originating from asymptomatic patients/staff members. Higher risk countries adopted more preventive measures including universal testing, routine testing of hospital staff and use of dedicated personal protective equipment in operation theatres, but there were remarkable discrepancies across the countries.

**Conclusions:**

This large international survey captured the global surgical practice under the COVID-19 pandemic and highlighted the insufficient preoperative screening of COVID-19 in the current surgical practice. More intensive screening programs will be necessary particularly in severely affected countries/institutions.

**Study registration:**

Registered in ClinicalTrials.gov: NCT04344197

The global pandemic of Coronavirus Disease 2019 (COVID-19) was announced by the World Health Organization (WHO) on 11 March, 2020 [1] and as of April 17, 2020, more than 2.2 million cases and more than 140 000 deaths have been reported in 210 countries.[2] The rapid spread of the outbreak has changed the global health care system, including the field of surgery: currently, many hospitals are forced to stop or postpone elective surgical interventions [3-5].

Patients infected by COVID-19 may present without typical symptoms [6,7] such as fever, cough, shortness of breath, gastrointestinal symptoms [8-10] anosmia and ageusia. Such asymptomatic patients play an important role in the disease spread [11-14]. In a surgical setting, asymptomatic COVID-19 patients may potentially expose health care providers to virus-contaminated aerosol through surgical and anesthetic procedures, transmit the disease to other hospitalized patients and trigger in-hospital outbreaks [15-17]. Furthermore, it was reported that COVID-19 patients have worse postoperative outcomes [18-20] with an unexpectedly high morbidity and mortality, reaching 44% Intensive Care Unit (ICU) admission and 20.5% deaths [21], possibly due to the postoperative suppression of cell-mediated immunity [22-24]. Despite these risks for health care workers, other patients, and the COVID-19 patients themselves, there have been no consistent statements on surgical guidelines [25-30] regarding the perioperative screening or management of COVID-19 patients, and we do not have objective global data that describe the current conditions surrounding this issue.

This international survey aimed to clarify the current global situation of surgical practice including COVID-19 screening, preventive measures and in-hospital infection under the COVID-19 pandemic, and to clarify the international/institutional gaps on infection control policies among countries worldwide.

## METHODS

### Study design

A cross-sectional online survey study on surgical practices was conducted in April, 2020 [31]. The survey questionnaires were designed and developed by the steering committee composed of 5 surgeons (VB, TK, YA, GP, GSS) through international teleconferences and email exchanges. As the COVID-19 pandemic was an unprecedented event, there were no referable previous surveys during this process. A pilot version of the survey was circulated and tested by 47 participants between March 24 and 30, 2020, and the revised final version was approved by all the authors of this study on April 1st, 2020.

The survey consisted of itemized closed questions (single choice, multiple choice, and numeric) as shown in **Table 1**. Main outcome and measures included centers’ experiences of in-hospital COVID-19 infections, and preventive measures and screening policies of COVID-19 in surgical practices. All questions were mandatory, and the participants were asked to provide profiles and names of the institutions to exclude duplicated registration.

**Table 1:** Survey questions

Basic information
Your country
Your city
Name of your hospital
Your center is? (type of hospital)
Have any COVID-19 positive patients been admitted to your hospitals? (both medicine and surgery department, with number of caseloads)
**Surgical procedures and protective measures (tables 2-4)**
To date, which kind of surgical procedures do you still perform at your hospital?
How do you perform surgery?
When is a surgical patient isolated in your center?
Are you doing hospital team-rotations at your center? (e.g.: divide department staff member in two separated groups that works separately on rotation)
When do you wear medical masks?
Which of the following are easily available at your hospital?
**Testing policies and In-hospital COVID-19 infection (tables 5-7)**
If local guidelines are available at your center, which surgical patients do your local guidelines recommend testing?
Do you perform a diagnostic Chest CT scan preoperatively to rule out COVID-19?
When are hospital staff members tested at your hospital?
Which type of test do you perform?
How long does it take to get COVID-19 test results on average at your center?
Have you experienced any in-hospital COVID-19 infections in your center?
If yes, did you manage to trace the outbreak?
Did any of your staff develop symptoms and test positive for COVID-19?
If any hospital staff member is tested positive while being asymptomatic, they:

The survey respondents were surgeons who represented the centers’ surgical departments, and the individual surgeons were responsible for providing data on surgical practice at the centers. The centers in this study included academic hospitals, cancer centers and local public or private hospitals that were equipped with surgical departments. Survey participation was on a voluntary basis. In light of the rapidly changing situation in each country, we conducted the online survey within one week (April 2 to 8, 2020). Google Forms (Google LLC, Menlo Park California, USA) was used to deliver the survey in 13 languages, and the Wenjuanxing platform (Changsha Ranxing Information Technology Co.,LTD, Hunan, China) was used to deliver a Chinese version in China. The survey was globally distributed to surgeons through emails, telephones, social media, and international surgical societies’ social media platforms (European Society of Surgical Oncology, Latin American Society of Surgical Oncology, Russian Society of Colorectal Surgeons and Società Italiana di Chirurgia Colo-Rettale).

The study was approved by the institutional review board of Tor Vergata University of Rome (n.49/20). The study was registered on ClinicalTrials.gov (NCT04344197). Reporting of this study follows the American Association for Public Opinion Research reporting guideline and Checklist for Reporting Results of Internet E-Surveys [32].

### Statistical analyses

**Figure 1:**
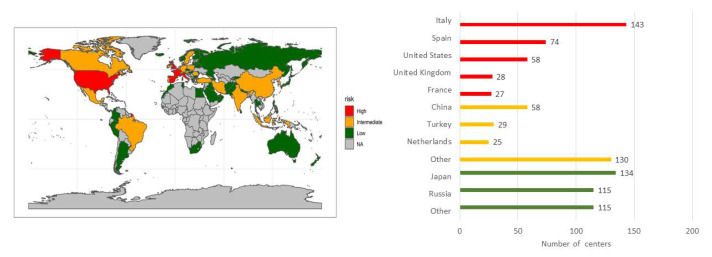
Country’s risk category and number of centers. Countries were classified into high (>5000), intermediate (100-5000) and low (<100) risk groups based on the number of cumulative deaths reported by the WHO on April 8, 2020. Other countries in intermediate risk (n = 130): Mexico (24), Portugal (17), Romania (13), Austria (12), Germany, India, Ireland (10), Belgium (8), Brazil, Switzerland (5), Canada, Sweden (4), South Korea (3), Indonesia (2), Denmark, Iran, and Poland (1). Other countries in low risk (n = 115): Ukraina (11), Azerbaijan (9), Pakistan (8), Egypt, Israel (6), Greece, Lithuania, Norway (5), Belarus, Colombia, Lebanon (4), Australia, Czech Republic, Sri Lanka (3), Argentina, Armenia, Bulgaria, Finland, Latvia, Moldova, Puerto Rico, South Africa, Thailand (2), Afghanistan, Hungary, Iceland, Iraq, Israel, Korea, Kyrgyzstan, Morocco, Nepal, New Zealand, North Macedonia, Oman, Palestine, Panama, Perú, Philippines, Saudi Arabia, Serbia, Singapore, Tunisia, United Arab Emirates (1).

Data were analyzed by country’s risk category. Countries were classified into high (>5000), intermediate (between 100 and 5000) and low (<100) risk groups based on the number of cumulative deaths reported by the WHO on April 8, 2020 (**Figure 1**). These death thresholds were defined by the fact that the most severely affected countries reached over 5000 deaths, and most countries started lockdown when the deaths exceeded 100 [33]. Data from countries with ≥25 centers were separately analyzed. Data were also analyzed by the centers’ COVID-19 caseloads (>100, 50-100, 10-50, 1-10 and none). Only one representative respondent per center was included in the analyses, and duplicated registration from the same centers was manually excluded based on the provided profiles and names of the institutions. Comparison of the data was performed using the χ^2^ and Fisher exact tests for categorical variables and the *t* test for continuous variables. Two-sided *P* values <0.05 were considered statistically significant. Analyses were performed by a statistician (AP) using R software (R Core Team (2019). R: A language and environment for statistical computing (R Foundation for Statistical Computing, Vienna, Austria).

## RESULTS

### Study population

The survey was completed by a total of 1173 surgeons from 936 centers in 71 countries, involving 5 high risk countries (330 centers), 20 intermediate risk countries (242 centers) and 46 low risk countries (364 centers) (**Figure 1**). Five high risk countries (Italy, Spain, USA, UK and France), 3 intermediate risk countries (China, Turkey, Netherlands) and 2 low risk countries (Japan and Russia) had 25 or more participating centers. There were 201 COVID-19-free, 59 COVID-19-dedicated and 642 COVID-19-mixed hospitals. The Types of centers were 342 academic centers, 155 cancer centers and 435 local public or private centers. Centers’ COVID-19 caseloads were available in 813 centers (86.9%).

### Surgical procedures and protective measures

Results of current surgical procedures and protective measures by countries’ risk group, country, and centers’ COVID-19 caseloads are summarized in **Table 2**, **Table 3** and **Table 4**. Overall, the majority of the centers performed emergency surgery (92.2%) and oncologic elective surgeries (68.4%), whereas non-oncologic elective surgeries were performed in 28.1% of the centers, ranging from 8.5% in high-risk to 45.1% in low-risk countries. Among the high risk countries, centers that continue oncologic elective surgeries varied from 48.3% in the USA to >80% in Italy and France. Centers that avoided laparoscopic surgery were less than 30% across countries in each risk category, except for UK (64.3%) and Turkey (51.7%). The use of dedicated Personal Protective Equipment (PPE) and the use of smoke-aspiration devices during laparoscopic surgery were proportional to the country risk categories and centers’ caseloads. Over 30% of the centers in high and intermediate risk countries organized hospital team rotation, but the proportion per each country varied from 17.5% (Italy) to 69% (Turkey). Overall, 71.8% of the participants reported always wearing a medical mask in the hospital, but the proportion varied across the countries from 0% in the Netherlands, 21.4% in UK to >95% in Italy, France and China. Among PPEs, FFP2/FFP3 masks and eye protections were less available similarly across the countries.

**Table 2:** Surgical procedures and protective measures by countries’ risk group

	Overall	Countries’ risk group			
**High risk**	**Int. risk**	**Low risk**	***P* value**
**936**	**330**	**242**	**364**	
Surgical procedures performed. No. (%):*					
Emergency	863 (92.2)	317 (96.1)	230 (95.0)	316 (86.8)	<0.001
Oncologic elective	640 (68.4)	222 (67.3)	189 (78.1)	229 (62.9)	<0.001
Non oncologic elective	263 (28.1)	28 (8.5)	71 (29.3)	164 (45.1)	<0.001
Office procedures and one-day surgery	159 (17.0)	17 (5.2)	41 (16.9)	101 (27.8)	<0.001
How do you perform surgery? No. (%):*					
As usual	398 (42.5)	75 (22.7)	93 (38.4)	230 (63.2)	<0.001
Try to avoid laparoscopic cases	187 (20.0)	79 (23.9)	63 (26.0)	45 (12.4)	<0.001
With dedicated PPE	320 (34.2)	178 (53.9)	74 (30.6)	68 (18.7)	<0.001
If laparoscopic, use smoke aspiration devices	238 (25.4)	133 (40.3)	57 (23.6)	48 (13.2)	<0.001
**When is a surgical patient isolated? No. (%)**					
Every patient is isolated until proved COVID-19 negative	101 (10.8)	62 (18.8)	27 (11.2)	12 (3.3)	<0.001
If symptomatic/suspected/COVID-19 positive	722 (77.1)	258 (78.2)	202 (83.5)	262 (72.0)	
Others	113 (12.1)	10 (3.0)	13 (5.4)	90 (24.7)	
**Are you doing hospital team-rotations? No. (%):**					
Yes	268 (28.6)	105 (31.8)	96 (39.7)	67 (18.4)	<0.001
No	668 (71.4)	225 (68.2)	146 (60.3)	297 (81.6)	
When do you wear medical masks? No. (%):*					
When visiting symptomatic/suspected/COVID-19 positive patients	141 (15.1)	40 (12.1)	48 (19.8)	53 (14.6)	0.04
When visiting every patient	124 (13.2)	34 (10.3)	29 (12.0)	61 (16.8)	0.04
Always in hospital	677 (71.8)	265 (80.3)	159 (65.7)	253 (69.5)	<0.001
Which are easily available: No. (%):*					
Gloves	880 (94.0)	309 (93.6)	227 (93.8)	344 (94.5)	0.88
Gowns	573 (61.3)	186 (56.4)	130 (53.9)	257 (70.6)	<0.001
Eye protection	405 (43.3)	121 (36.7)	113 (46.7)	171 (47.0)	0.01
Medical masks	736 (78.6)	259 (78.5)	196 (81.0)	281 (77.2)	0.54
FFP2/FFP3 or respirator N95	231 (24.7)	83 (25.2)	71 (29.3)	77 (21.2)	0.07
Hand sanitizer	791 (84.5)	263 (79.7)	216 (89.3)	312 (85.7)	0.005

Int – intermediate, PPE – personal protective equipment

*Percentages do not add up to 100 because of the multiple choice questions.

**Table 3:** Surgical procedures and protective measures by country (countries with ≥25 centers**)**

	Overall			Countries																				
**Italy-H**			**Spain-H**		**USA-H**		**UK-H**		**France-H**		**China-I**		**Turkey-I**		**Netherlands-I**		**Japan-L**		**Russia-L**	
936			143			74		58		28		27		58		29		25		134		115	
Surgical procedures performed. No. (%):*																								
Emergency	863 (92.2)			132 (92.3)			73 (98.6)		58 (100.0)		28 (100.0)		26 (96.3)		54 (93.1)		28 (96.6)		25 (100.0)		120 (89.6)		84 (73.0)	
Oncologic elective	640 (68.4)			115 (80.4)			42 (56.8)		28 (48.3)		15 (53.6)		22 (81.5)		55 (94.8)		24 (82.8)		24 (96.0)		131 (97.8)		35 (30.4)	
Non oncologic elective	263 (28.1)			16 (11.2)			0 (0.0)		7 (12.1)		1 (3.6)		4 (14.8)		42 (72.4)		4 (13.8)		3 (12.0)		111 (82.8)		45 (39.1)	
Office procedures and one-day surgery	159 (17.0)			7 (4.9)			0 (0.0)		9 (15.5)		1 (3.6)		0 (0.0)		24 (41.4)		1 (3.4)		1 (4.0)		68 (51.1)		24 (20.9)	
How do you perform surgery? No. (%):*																								
As usual	398 (42.5)			36 (25.2)			9 (12.2)		19 (32.8)		2 (7.1)		9 (33.3)		36 (62.1)		5 (17.2)		12 (48.0)		119 (88.8)		67 (58.3)	
Try to avoid laparoscopic cases	187 (20.0)			30 (21.0)			21 (28.4)		6 (10.3)		18 (64.3)		4 (14.8)		7 (12.1)		15 (51.7)		1 (4.0)		3 (2.2)		7 (6.1)	
With dedicated PPE	320 (34.2)			77 (53.8)			47 (63.5)		24 (41.4)		20 (71.4)		10 (37.0)		14 (24.1)		13 (44.8)		4 (16.0)		1 (0.7)		34 (29.6)	
If laparoscopic, use smoke aspiration devices	238 (25.4)			48 (33.6)			46 (62.2)		18 (31.0)		7 (25.0)		14 (51.9)		14 (24.1)		2 (6.9)		13 (52.0)		30 (22.4)		0 (0.0)	
**When is a surgical patient isolated? No. (%):**																								
Every patient is isolated until proved COVID-19 negative	101 (10.8)			35 (24.5)			14 (18.9)		8 (13.8)		1 (3.6)		4 (14.8)		9 (15.5)		2 (6.9)		0 (0.0)		2 (1.5)		1 (0.9)	
If symptomatic/ suspected/ COVID-19 positive	722 (77.1)			102 (71.3)			56 (75.7)		50 (86.2)		26 (92.9)		23 (85.2)		45 (77.6)		26 (89.7)		25 (100.0)		116 (86.6)		57 (49.6)	
Others	113 (12.1)			6 (4.2)			4 (5.4)		0 (0.0)		1 (3.6)		0 (0.0)		4 (6.9)		1 (3.4)		0 (0.0)		16 (11.9)		57 (49.6)	
**Are you doing hospital team-rotations? No. (%):**																								
Yes		268 (28.6)		25 (17.5)			39 (52.7)		22 (37.9)		13 (46.4)		6 (22.2)		18 (31.0)		20 (69.0)		8 (32.0)		5 (3.7)		11 (9.6)	
No		668 (71.4)		118 (82.5)			35 (47.3)		36 (62.1)		15 (53.6)		21 (77.8)		40 (69.0)		9 (31.0)		17 (68.0)		129 (96.3)		104 (90.4)	
When do you wear medical masks? No. (%):*																							
When visiting symptomatic/ suspected/ COVID-19 positive patients		141 (15.1)			0 (0.0)	12 (16.2)		13 (22.4)		14 (50.0)		1 (3.7)		1 (1.7)		4 (13.8)		18 (72.0)		13 (9.7)		10 (8.7)	
When visiting every patient		124 (13.2)			4 (2.8)	11 (14.9)		8 (13.8)		11 (39.3)		0 (0.0)		1 (1.7)		6 (20.7)		3 (12.0)		23 (17.2)		22 (19.1)	
Always in hospital		677 (71.8)			136 (95.1)	60 (81.1)		37 (63.8)		6 (21.4)		26 (96.3)		56 (96.6)		19 (65.5)		0 (0.0)		104 (77.6)		77 (67.0)	
Which are easily available: No. (%):*																							
Gloves		880 (94.0)	136 (95.1)			71 (95.9)		50 (86.2)		28 (100.0)		24 (88.9)		52 (89.7)		29 (100.0)		25 (100.0)		125 (93.3)		111 (96.5)	
Gowns		573 (61.3)	65 (45.5)			44 (59.5)		41 (70.7)		23 (82.1)		13 (48.1)		19 (32.8)		15 (51.7)		23 (95.8)		105 (78.4)		77 (67.0)	
Eye protection		405 (43.3)	48 (33.6)			22 (29.7)		35 (60.3)		11 (39.3)		5 (18.5)		27 (46.6)		12 (41.4)		23 (92.0)		77 (57.5)		45 (39.1)	
Medical masks		736 (78.6)	118 (82.5)			47 (63.5)		45 (77.6)		23 (82.1)		26 (96.3)		51 (87.9)		24 (82.8)		25 (100.0)		95 (70.9)		97 (84.3)	
FFP2/FFP3 or respirator N95		231 (24.7)	30 (21.0)			19 (25.7)		22 (37.9)		9 (32.1)		3 (11.1)		12 (20.7)		9 (31.0)		15 (60.0)		38 (28.4)		8 (7.0)	
Hand sanitizer		791 (84.5)	109 (76.2)			66 (89.2)		47 (81.0)		20 (71.4)		21 (77.8)		55 (94.8)		25 (86.2)		25 (100.0)		107 (79.9)		105 (91.3)	

Country-H – High risk country, Country-I – Intermediate risk country, Country-L – Low risk country, PPE – personal protective equipment

*Percentages do not add up to 100 because of the multiple choice questions.

**Table 4:** Surgical procedures and protective measures by centers’ COVID-19 caseloads

	Overall	Centers’ COVID-19 caseloads					
**>100**	**50-100**	**10-50**	**1-10**	**None**	***P* value**
**813**	**32**	**177**	**151**	**222**	**231**	
Surgical procedures performed. No. (%):*							
Emergency	746 (91.8)	30 (93.8)	173 (97.7)	147 (97.4)	205 (92.3)	191 (82.7)	<0.001
Oncologic elective	575 (70.7)	21 (65.6)	139 (78.5)	116 (76.8)	160 (72.1)	139 (60.2)	<0.001
Non oncologic elective	246 (30.3)	1 (3.1)	18 (10.2)	33 (21.9)	84 (37.8)	110 (47.6)	<0.001
Office procedures and one-day surgery	149 (18.3)	2 (6.2)	10 (5.6)	17 (11.3)	54 (24.4)	66 (28.6)	<0.001
How do you perform surgery? No. (%):*							
As usual	367 (45.1)	6 (18.8)	44 (24.9)	65 (43.0)	111 (50.0)	141 (61.0)	<0.001
Try to avoid laparoscopic cases	162 (19.9)	10 (31.2)	40 (22.6)	37 (24.5)	46 (20.7)	29 (12.6)	0.01
With dedicated PPE	273 (33.6)	23 (71.9)	97 (54.8)	55 (36.4)	55 (24.8)	43 (18.6)	<0.001
If laparoscopic, use smoke aspiration devices	212 (26.1)	17 (53.1)	78 (44.1)	47 (31.1)	41 (18.5)	29 (12.6)	<0.001
**When is a surgical patient isolated? No. (%):**							
Every patient is isolated until proved COVID-19 negative	90 (11.1)	5 (15.6)	41 (23.2)	23 (15.2)	13 (5.9)	8 (3.5)	<0.001
If symptomatic/ suspected/ COVID-19 positive	622 (76.5)	26 (81.2)	133 (75.1)	126 (83.4)	192 (86.5)	150 (64.9)	
Others	101 (12.4)	1 (3.1)	3 (1.7)	2 (1.3)	17 (7.7)	73 (31.6)	
**Are you doing hospital team-rotations? No. (%):**							
Yes	234 (28.8)	10 (31.2)	76 (42.9)	52 (34.4)	53 (23.9)	43 (18.6)	<0.001
No	579 (71.2)	22 (68.8)	101 (57.1)	99 (65.6)	169 (76.1)	188 (81.4)	
When do you wear medical masks? No. (%)*							
When visiting symptomatic/ suspected/ COVID-19 positive patients	125 (15.4)	2 (6.2)	33 (18.6)	31 (20.5)	31 (14.0)	28 (12.1)	0.07
When visiting every patient	109 (13.4)	6 (18.8)	17 (9.6)	20 (13.2)	28 (12.6)	38 (16.5)	0.29
Always in hospital	590 (72.6)	26 (81.2)	128 (72.3)	106 (70.2)	165 (74.3)	165 (71.4)	0.71
Which are easily available: No. (%):*							
Gloves	764 (94.0)	30 (93.8)	170 (96.0)	141 (93.4)	207 (93.2)	216 (93.5)	0.78
Gowns	499 (61.4)	22 (68.8)	107 (60.8)	89 (58.9)	138 (62.2)	143 (61.9)	0.86
Eye protection	363 (44.6)	13 (40.6)	83 (46.9)	60 (39.7)	105 (47.3)	102 (44.2)	0.61
Medical masks	640 (78.7)	25 (78.1)	146 (82.5)	118 (78.1)	171 (77.0)	180 (77.9)	0.74
FFP2/FFP3 or respirator N95	201 (24.7)	11 (34.4)	55 (31.1)	45 (29.8)	48 (21.6)	42 (18.2)	0.007
Hand sanitizer	707 (87.0)	24 (75.0)	154 (87.0)	133 (88.1)	199 (89.6)	197 (85.3)	0.19

PPE – personal protective equipment

*Percentages do not add up to 100 because of the multiple choice questions. Data were analyzed in centers that provided COVID-19 caseloads (N = 813).

### Testing policies

Testing policies by countries’ risk group, country, and centers’ COVID-19 caseloads are summarized in in **Table 5**, **Table 6** and **Table 7**. The majority (71.9%) of local guidelines recommended preoperative testing based on symptoms or suspicious radiologic findings. Local guidelines recommended testing every surgical patient in less than 20% of the centers. Routine screening by chest-Computer Tomography (CT) scan was used in only 22.8% of the overall centers, and the rates varied among the countries from 87.9% in China to 7.3% in the USA. Testing policies for staff members were also based on symptoms or risk contact in majority of the centers. Polimerase Chain Reaction (PCR) test without antibody testing was used in most of the countries, whereas nearly 30% of surgeons did not know which type of laboratory testing was used at their centers. The wait time to get test results was more than 1 day in 34.7% of the centers.

**Table 5:** Testing policies and in-hospital COVID-19 infection by countries’ risk group

	Overall	Countries’ risk group			
**High risk**	**Int. risk**	**Low risk**	***P* value**
936	330	242	364	
Testing policies recommended by the local guidelines. No. (%):*					
Everyone	172 (18.4)	84 (25.5)	54 (22.3)	34 (9.3)	<0.001
All oncologic patients	56 (6.0)	32 (9.7)	13 (5.4)	11 (3.0)	0.001
All emergency patients	101 (10.8)	40 (12.1)	27 (11.2)	34 (9.3)	0.49
Symptomatic or suspicious radiological features	673 (71.9)	222 (67.3)	164 (67.8)	287 (78.8)	0.04
**Preoperative chest CT performed. No. (%):**					
Yes	213 (22.8)	81 (24.5)	88 (36.4)	44 (12.1)	<0.001
No	394 (42.1)	144 (43.6)	88 (36.4)	162 (44.5)	
Only if symptomatic	320 (34.2)	100 (30.3)	66 (27.3)	154 (42.3)	
Others	9 (1.0)	5 (1.5)	0 (0.0)	4 (1.1)	
Testing policies on staff members. No. (%):*					
Everyone is routinely tested every two/four weeks	23 (2.5)	14 (4.2)	2 (0.8)	7 (1.9)	0.02
Mandatory if risk contact/symptoms present	570 (60.9)	186 (56.4)	157 (64.9)	227 (62.4)	0.09
On request if risk contact/symptoms present	435 (46.5)	174 (52.7)	122 (50.4)	139 (38.2)	<0.001
No test	46 (4.9)	14 (4.2)	8 (3.3)	24 (6.6)	0.15
**Testing type. No. (%):**					
1 PCR swab	392 (41.9)	186 (56.4)	81 (33.5)	125 (34.3)	<0.001
2 PCR swabs	216 (23.1)	75 (22.7)	67 (27.7)	74 (20.3)	
PCR + antibodies	71 (7.6)	16 (4.8)	36 (14.9)	19 (5.2)	
I don't know	257 (27.5)	53 (16.1)	58 (24.0)	146 (40.1)	
**Wait time for test results. No. (%):**					
1-6 h	169 (18.1)	63 (19.1)	46 (19.0)	60 (16.5)	<0.001
6 h-1 d	305 (32.6)	126 (38.2)	82 (33.9)	97 (26.6)	
More than 1 d	325 (34.7)	126 (38.2)	84 (34.7)	115 (31.6)	
I don't know	137 (14.6)	15 (4.5)	30 (12.4)	92 (25.3)	
**Experienced in-hospital COVID-19 infection. No. (%):**					
Yes	295 (31.5)	177 (53.6)	64 (26.4)	54 (14.8)	<0.001
No/I don’t know	641 (68.5)	153 (46.4)	178 (73.6)	310 (85.2)	
**If yes, source of the outbreak traced? No. (%):†**					
We had hospital outbreak but could not trace them	122 (41.4)	92 (52.0)	18 (28.1)	12 (22.2)	<.001
Yes, started from a symptomatic staff/patient	89 (30.2)	42 (23.7)	27 (42.2)	20 (37.0)	
Yes, started from an asymptomatic staff/patient	58 (19.7)	31 (17.5)	14 (21.9)	13 (24.1)	
Others	26 (8.8)	12 (6.8)	5 (7.8)	9 (16.7)	
**Staff testing positive with symptoms. No. (%):**					
Yes	296 (31.6)	162 (49.1)	89 (36.8)	45 (12.4)	<.001
No/I don’t know	640 (68.4)	168 (50.9)	153 (63.2)	319 (87.6)	
Policies for asymptomatic infected staff. No. (%):*					
Placed in mandatory quarantine	725 (77.5)	249 (75.5)	195 (80.6)	281 (77.2)	.35
Placed in voluntary quarantine	125 (13.4)	40 (12.1)	30 (12.4)	55 (15.1)	.45
Continue working	41 (4.4)	25 (7.6)	7 (2.9)	9 (2.5)	.002

Int – intermediate, CT – computed tomography, PCR – polymerase chain reaction

*Percentages do not add up to 100 because of the multiple choice questions.

†The sum was the number of the respondents who answered “yes” in the question “Experienced in-hospital COVID-19 infection”.

**Table 6:** Testing policies and in-hospital COVID-19 infection by country (countries with ≥25 centers)

	Overall			Countries																		
**Italy-H**		**Spain-H**		**USA-H**		**UK-H**		**France-H**		**China-I**		**Turkey-I**		**Netherlands-I**		**Japan-L**		**Russia-L**
936			143		74		58		28		27		58		29		25		134		115
Testing policies recommended by the local guidelines. No. (%):*																						
Everyone	172 (18.4)			45 (31.5)		21 (28.4)		7 (12.1)		5 (17.9)		6 (22.2)		11 (19.0)		5 (17.2)		10 (40.0)		10 (7.5)		6 (5.2)
All oncologic patients	56 (6.0)			17 (11.9)		8 (10.8)		1 (1.7)		4 (14.3)		2 (7.4)		0 (0.0)		4 (13.8)		1 (4.0)		4 (3.0)		2 (1.7)
All emergency patients	101 (10.8)			20 (14.0)		10 (13.5)		2 (3.4)		4 (14.3)		4 (14.8)		5 (8.6)		5 (17.2)		3 (12.0)		7 (5.2)		7 (6.1)
Symptomatic or suspicious radiological features	673 (71.9)			89 (62.2)		47 (63.5)		43 (74.1)		21 (75.0)		22 (81.5)		46 (79.3)		20 (69.0)		13 (52.0)		115 (85.8)		89 (77.4)
**Preoperative chest CT performed. No. (%):**																						
Yes	213 (22.8)			35 (24.5)		20 (27.0)		4 (6.9)		14 (50.0)		8 (29.6)		51 (87.9)		13 (44.8)		11 (44.0)		22 (16.4)		6 (5.2)
No	394 (42.1)			51 (35.7)		41 (55.4)		37 (63.8)		8 (28.6)		7 (25.9)		2 (3.4)		3 (10.3)		5 (20.0)		40 (29.9)		68 (59.1)
Only if symptomatic	320 (34.2)			56 (39.2)		12 (16.2)		14 (24.1)		6 (21.4)		12 (44.4)		5 (8.6)		13 (44.8)		9 (36.0)		71 (53.0)		38 (33.0)
Others	9 (1.0)			1 (0.7)		1 (1.4)		3 (5.2)		0 (0)		0 (0)		0 (0)		0 (0)		0 (0)		1 (0.7)		3 (2.6)
Testing policies on staff members. No. (%):*																						
Everyone is routinely tested every two/four weeks	23 (2.5)			13 (9.1)		1 (1.4)		0 (0.0)		0 (0.0)		0 (0.0)		0 (0.0)		0 (0.0)		0 (0.0)		1 (0.7)		4 (3.5)
Mandatory if risk contact/symptoms	570 (60.9)			109 (76.2)		35 (47.3)		24 (41.4)		5 (17.9)		13 (48.1)		29 (50.0)		24 (82.8)		19 (76.0)		81 (60.4)		74 (64.3)
On request if risk contact/symptoms	435 (46.5)			51 (35.7)		49 (66.2)		38 (65.5)		17 (60.7)		19 (70.4)		37 (63.8)		12 (41.4)		6 (24.0)		64 (47.8)		16 (13.9)
No test	46 (4.9)			1 (0.7)		0 (0.0)		7 (12.1)		5 (17.9)		1 (3.7)		1 (1.7)		0 (0.0)		1 (4.0)		0 (0.0)		19 (16.5)
**Testing type. No. (%):**																						
1 PCR swab	392 (41.9)			74 (51.7)		47 (63.5)		33 (56.9)		14 (50.0)		18 (66.7)		6 (10.3)		7 (24.1)		12 (48.0)		53 (39.6)		23 (20.0)
2 PCR swabs	216 (23.1)			41 (28.7)		15 (20.3)		6 (10.3)		8 (28.6)		5 (18.5)		17 (29.3)		11 (37.9)		9 (36.0)		12 (9.0)		32 (27.8)
PCR + antibodies	71 (7.6)			13 (9.1)		2 (2.7)		0 (0.0)		0 (0.0)		1 (3.7)		23 (39.7)		4 (13.8)		0 (0.0)		4 (3.0)		9 (7.8)
I don't know	257 (27.5)			15 (10.5)		10 (13.5)		19 (32.8)		6 (21.4)		3 (11.1)		12 (20.7)		7 (24.1)		4 (16.0)		65 (48.4)		51 (44.3)
**Wait time for test results. No. (%):**																						
1-6 h	169 (18.1)			31 (21.7)		20 (27.0)		2 (3.4)		3 (10.7)		7 (25.9)		17 (29.3)		2 (6.9)		11 (44.0)		18 (13.4)		9 (7.8)
6 h-1 d	305 (32.6)			71 (49.7)		30 (40.5)		6 (10.3)		7 (25.0)		12 (44.4)		16 (27.6)		4 (13.8)		13 (52.0)		46 (34.3)		13 (11.3)
More than 1 d	325 (34.7)			35 (24.5)		22 (29.7)		46 (79.3)		15 (53.6)		8 (29.6)		8 (13.8)		18 (62.1)		1 (4.0)		35 (26.1)		48 (41.7)
I don't know	137 (14.6)			6 (4.2)		2 (2.7)		4 (6.9)		3 (10.7)		0 (0.0)		17 (29.3)		5 (17.2)		0 (0.0)		35 (26.1)		45 (39.1)
**Experienced in-hospital COVID-19 infection. No. (%):**																						
Yes	295 (31.5)			93 (65.0)		47 (63.5)		11 (19.0)		13 (46.4)		13 (48.1)		2 (3.4)		12 (41.4)		14 (56.0)		11 (8.2)		20 (17.4)
No/I don’t know	641 (68.5)			50 (35.0)		27 (36.5)		47 (81.0)		15 (53.6)		14 (51.9)		56 (96.6)		17 (58.6)		11 (44.0)		123 (91.8)		95 (82.6)
**If yes, source of the outbreak traced? No. (%):†**																							
We had hospital outbreak but could not trace them		122 (41.4)	48 (51.6)		27 (57.4)		2 (18.2)		7 (53.8)		8 (61.5)		1 (50.0)		0 (0.0)		5 (35.7)		2 (18.2)		8 (40.0)		
Yes, started from a symptomatic staff member/patient		89 (30.2)	21 (22.6)		9 (19.1)		5 (45.5)		3 (23.1)		4 (30.8)		1 (50.0)		8 (66.7)		6 (42.9)		1 (9.1)		8 (40.0)		
Yes, started from an asymptomatic staff member/patient		58 (19.7)	22 (23.7)		7 (14.9)		0 (0.0)		1 (7.7)		1 (7.7)		0 (0.0)		4 (33.3)		2 (14.3)		0 (0.0)		4 (20.0)		
Others		26 (8.8)	2 (2.2)		4 (8.5)		4 (36.4)		2 (15.4)		0 (0.0)		0 (0.0)		0 (0)		1 (7.1)		8 (72.7)		0 (0)		
**Staff testing positive with symptoms. No. (%):**																							
Yes		296 (31.6)	62 (43.4)		41 (55.4)		25 (43.1)		17 (60.7)		17 (63.0)		6 (10.3)		15 (51.7)		18 (72.0)		9 (6.7)		9 (7.8)		
No/I don’t know		640 (68.4)	81 (56.6)		33 (44.6)		33 (56.9)		11 (39.3)		10 (37.0)		52 (89.7)		14 (48.3)		7 (28.0)		125 (93.3)		106 (92.2)		
Policies for asymptomatic infected staff. No. (%):*																							
Placed in mandatory quarantine		725 (77.5)	117 (81.8)		62 (83.8)		35 (60.3)		20 (71.4)		15 (55.6)		47 (81.0)		21 (72.4)		19 (76.0)		111 (82.8)		68 (59.1)		
Placed in voluntary quarantine		125 (13.4)	15 (10.5)		3 (4.1)		9 (15.5)		6 (21.4)		7 (25.9)		10 (17.2)		5 (17.2)		3 (12.0)		32 (23.9)		16 (13.9)		
Continue working		41 (4.4)	7 (4.9)		2 (2.7)		7 (12.1)		2 (7.1)		7 (25.9)		0 (0.0)		0 (0.0)		0 (0.0)		1 (0.7)		6 (5.2)		

Country-H - High risk country, Country-I - Intermediate risk country, Country-L - Low risk country, CT - computed tomography, PCR - polymerase chain reaction

*Percentages do not add up to 100 because of the multiple choice questions.

†The sum was the number of the respondents who answered “yes” in the question “Experienced in-hospital COVID-19 infection.”

**Table 7:** Testing policies and in-hospital COVID-19 infection by centers’ COVID-19 caseloads

	Overall	Centers’ COVID-19 caseloads					
**>100**	**50-100**	**10-50**	**1-10**	**None**	***P* value**
813	32	177	151	222	231	
Testing policies recommended by the local guidelines. No. (%)*							
Everyone	155 (19.1)	22 (68.8)	52 (29.4)	27 (17.9)	28 (12.6)	26 (11.3)	<0.001
All oncologic patients	49 (6.0)	3 (9.4)	18 (10.2)	13 (8.6)	5 (2.3)	10 (4.3)	0.006
All emergency patients	88 (10.8)	4 (12.5)	22 (12.4)	23 (15.2)	20 (9.0)	19 (8.2)	0.20
Symptomatic or suspicious radiological features	580 (71.3)	6 (18.8)	108 (61.0)	115 (76.2)	177 (79.7)	174 (75.3)	<0.001
**Preoperative chest CT performed. No. (%)**							
Yes	184 (22.6)	13 (40.6)	44 (24.9)	41 (27.2)	44 (19.8)	42 (18.2)	0.14
No	339 (41.7)	10 (31.2)	68 (38.4)	64 (42.4)	94 (42.3)	103 (44.6)	
Only if symptomatic	282 (34.7)	9 (28.1)	64 (36.2)	46 (30.5)	81 (36.5)	82 (35.5)	
Others	8 (1.0)	0 (0)	1 (0.6)	0 (0)	3 (1.4)	4 (1.7)	
Testing policies on staff members. No. (%):*							
Everyone is routinely tested every two/four weeks	23 (2.8)	4 (12.5)	7 (4.0)	3 (2.0)	5 (2.3)	4 (1.7)	0.01
Mandatory if risk contact/symptoms	514 (63.2)	20 (62.5)	119 (67.2)	100 (66.2)	122 (55.0)	153 (66.2)	0.06
On request if risk contact/symptoms	381 (46.9)	12 (37.5)	92 (52.0)	87 (57.6)	115 (51.8)	75 (32.5)	<0.001
No test	34 (4.2)	1 (3.1)	5 (2.8)	3 (2.0)	10 (4.5)	15 (6.5)	0.21
**Testing type. No. (%):**							
1 PCR swab	338 (41.6)	24 (75.0)	95 (53.7)	64 (42.4)	89 (40.1)	66 (28.6)	<0.001
2 PCR swabs	189 (23.2)	6 (18.8)	48 (27.1)	38 (25.2)	45 (20.3)	52 (22.5)	
PCR + antibodies	62 (7.6)	1 (3.1)	8 (4.5)	12 (7.9)	18 (8.1)	23 (10.0)	
I don't know	224 (27.6)	1 (3.1)	26 (14.7)	37 (24.5)	70 (31.5)	90 (39.0)	
**Wait time for test results. No. (%)**							
1-6 h	148 (18.2)	10 (31.2)	39 (22.0)	35 (23.2)	33 (14.9)	31 (13.4)	<0.001
6 h-1 d	265 (32.6)	18 (56.2)	79 (44.6)	47 (31.1)	73 (32.9)	48 (20.8)	
More than 1 d	282 (34.7)	4 (12.5)	58 (32.8)	58 (38.4)	91 (41.0)	71 (30.7)	
I don't know	118 (14.5)	0 (0.0)	1 (0.6)	11 (7.3)	25 (11.3)	81 (35.1)	
**Experienced in-hospital COVID-19 infection. No. (%):**							
Yes	246 (30.3)	27 (84.4)	100 (56.5)	52 (34.4)	52 (23.4)	15 (6.5)	<0.001
No/I don’t know	567 (69.7)	5 (15.6)	77 (43.5)	99 (65.6)	170 (76.6)	216 (93.5)	
**If yes, source of the outbreak traced? No. (%):†**							
We had hospital outbreak but could not trace them	94 (38.2)	21 (77.8)	43 (43.0)	11 (21.2)	12 (23.1)	7 (46.7)	<0.001
Yes, started from a symptomatic staff member/patient	78 (31.7)	3 (11.1)	30 (30.0)	20 (38.5)	21 (40.4)	4 (26.7)	
Yes, started from an asymptomatic staff member/patient	53 (21.5)	3 (11.1)	22 (22.0)	14 (26.9)	11 (21.2)	3 (20.0)	
Others	21 (8.5)	0 (0)	5 (5.0)	7 (13.5)	8 (15.4)	1 (6.6)	
**Staff testing positive with symptoms. No. (%):**							
Yes	245 (30.1)	21 (65.6)	95 (53.7)	68 (45.0)	52 (23.4)	9 (3.9)	<0.001
No/I don’t know	568 (69.9)	11 (34.4)	82 (46.3)	83 (55.0)	170 (76.6)	222 (96.1)	
Policies for asymptomatic infected staff. No. (%):*							
Placed in mandatory quarantine	640 (78.7)	27 (84.4)	142 (80.2)	113 (74.8)	187 (84.2)	171 (74.0)	0.05
Placed in voluntary quarantine	111 (13.7)	2 (6.2)	18 (10.2)	24 (15.9)	36 (16.2)	31 (13.4)	0.27
Continue working	31 (3.8)	0 (0.0)	11 (6.2)	10 (6.6)	2 (0.9)	8 (3.5)	0.01

CT – computed tomography, PCR – polymerase chain reaction

*Percentages do not add up to 100 because of the multiple choice questions.

Data were analyzed in centers that provided COVID-19 caseloads (N = 813)

†The sum was the number of the respondents who answered “yes” in the question “Experienced in-hospital COVID-19 infection”.

### In-hospital COVID-19 infection

In-hospital COVID-19 infection by countries’ risk group, country, and centers’ COVID-19 caseloads are summarized in **Table 5**, **Table 6** and **Table 7**. Overall, in-hospital COVID-19 infection was reported in 31.5% of the overall centers, and the rate was highest in the high risk countries (53.6%), but some intermediate risk countries (Netherland, Turkey) also reported relatively high rates which were comparable to high risk countries. Out of 295 centers that experienced in-hospital COVID-19 infection, 122 (41.4%) failed to trace it, and 58 (19.7%) reported the infection originating from asymptomatic patients/staff members. When analyzed by institutional caseload of COVID-19 patients, centers that had treated high number of COVID-19 patients reported high rates of in-hospital COVID-19 infection and staff member infection.

## DISCUSSION

In this large international survey involving 936 centers from 71 countries, we revealed the current global situation of surgical practice including COVID-19 screening, preventive measures and in-hospital infection under the COVID-19 pandemic in early April 2020. To our knowledge, this is one of the largest international survey studies on COVID-19 in the field of surgery. The survey revealed two major findings. First, significant rates of centers had experienced in-hospital COVID-19 infection (31.5%) worldwide, and the majority of these centers failed to trace it or reported the infection originating from asymptomatic patients/staff members. The rates of in-hospital COVID-19 infection reached 53.6% in high risk counties and 84.4% in centers with >100 COVID-19 case load. Second, there were remarkable discrepancies among countries regarding the preoperative screening policies and perioperative preventive measures. We can conclude that under the current screening policies, COVID-19 patients impose problems with non-negligible frequency in surgical practice that may trigger hospital outbreaks, particularly in severely affected countries/institutions.

Since the early phase of COVID-19 pandemic, preventive measures and screening policies worldwide focused on symptomatic patients, mainly based on previous experience with the influenza virus and Corona Virus 1 Severe Acute Respiratory Syndrome (SARS-CoV-1). Our study was compatible with these findings, and confirmed wide prevalence of the initial symptom-based preoperative testing policies which may have missed the asymptomatic cases: less than 20% of local guidelines worldwide tested every surgical patient with huge variations among countries. Such limited use of preoperative testing may also be related to a worldwide shortage of testing capacity and to >1 day waiting periods for test results as reported from at least 34.5% of the centers.

Although we have no direct evidence on the benefit of universal testing for surgical patients, recent emerging evidence brought the international and local surgical guidelines to recommend preoperative testing when available and practical [25-30]. SARS-CoV-2 viral loads peak 5 days earlier than SARS-CoV-1, and is similarly detected in asymptomatic and symptomatic patients [34-36]. These traits of SARS-Cov-2 make asymptomatic patients more likely to transmit the disease than in the previous epidemics [37-39]. In hospital settings, a study from China reported a higher prevalence of asymptomatic COVID-19 infection in hospitalized patients (5.8%) compared to the community (1.2%),[40] and asymptomatic hospitalized patients were frequently reported as a source of in-hospital outbreaks [41]. In this study, local guidelines in the majority of the centers recommended testing based on symptoms or suspicious radiologic findings. Such testing policies may be challenged by the fact that over 30% of the centers worldwide experienced in-hospital COVID-19 infection, and almost 60% of those centers failed to trace it or reported originating from asymptomatic carriers. The proportion of centers with in-hospital COVID-19 infection was particularly high in severely affected countries/centers. In light of these findings, infection-control strategies focused solely on symptomatic patients may not be sufficient in surgical patients, particularly in highly affected countries/centers. A prospective universal testing program for surgical patients is warranted to clarify the prevalence of asymptomatic carriers, its potential impact on hospital outbreaks and the cost-benefit balance of the testing.

Another approach to deal with asymptomatic COVID-19 patients is the strict use of PPE and infection control measures. In this study, the universal use of dedicated PPE was proportional to the countries’ risk categories and centers’ caseloads, reaching 53.9% in the high risk countries and 71.9% in the highest caseload centers. This data implies surgeons’ high concern and awareness of asymptomatic COVID-19 patients in the highly-affected countries/centers. Unfortunately, the study also disclosed insufficient availability of the PPE across the countries, particularly Filter Face Piece2 (FFP)/FFP3 masks and eye protections. Large disparities existed in the availability of the PPE across the countries. Surgeons must consider the local testing capability as well as PPE availability to decide the best protective measures under the current risk of asymptomatic COVID-19 patients.

Policies on wearing a mask for health care workers have been debated. The WHO guidelines recommend that health care workers should wear a medical mask when entering a room where patients with suspected or confirmed COVID-19 are admitted [42]. Although 71.8% of the participants in this study reported that they always wore a mask in the hospital, the rates were not linearly correlated with countries’ risk category or centers’ caseload but varied significantly across the countries from 0% to >95%, which reflects the lack of international consensus and perhaps cultural differences on this subject. In contrast to mask policies, the use of dedicated PPE was linearly correlated with the country risk categories and centers’ caseloads. Interestingly, surgeons from UK reported the highest use of dedicated PPE in operation theatres (71.4%) but the lowest use of masks in hospital wards (21.4%) among the high risk countries. Further, our data showed that resource shortage was almost comparable in high caseload centers or high risk countries compared to the others. These findings suggest the use of PPE was dependent not only on supply but institutional or surgeons’ policies.

In this study, countries were classified into the 3 risk categories by the number of cumulative deaths in light of the epidemiological situations on April 8, 2020 as described in the Methods. Although there is no consensus about what is the best index for the countries’ pandemic status, this classification might be challenged as the number of deaths is not only dependent on the pandemic status but also on the population size and different definitions of the COVID-19-related deaths among the countries. Acknowledging these limitations, it is noteworthy that there were clear consistency between the results analyzed by the countries’ risk category and those analyzed by the centers’ COVID-19 caseload. For instance, centers with higher caseload (>100) adopted overall more preventive policies than lower caseload centers, including universal testing (in 100% of high caseload centers), routine testing of hospital staff and use of dedicated PPE in operation theatres. Similar trends were observed between these variables and the country risk category. The matching results between the two different risk categories (ie, countries’ risk category and centers’ caseloads) support the robustness of our analysis and results.

The strengths of this survey include large numbers and internationality of respondents and short period of recruitment to capture the current practice under rapid developments of COVID-19 crisis. However, this survey is subject to inherent systematic biases caused by unintended selection of centers at distribution, unequal number of centers per country and uneven geographical coverage. Only six countries (Italy, Spain, USA, China, Japan and Russia) were responsible for 62.2% of the participants, and the results may over-represent the situations in these countries. The response rate for the survey cannot be provided due to unlimited distribution through social media and academic societies, and the profiles or representativeness of the centers cannot be evaluated or compared between those did and did not respond to the survey. Voluntary participation of the survey may have resulted in recruiting respondents who have high interests in this topic and led to overestimation of the frequency and clinical impact of COVID-19 patients in surgical practice (a voluntary response bias). Acknowledging these limitations, this study tried to minimize the effect of such biases and obtain clinically meaningful results by collecting a large sample size and stratifying the data by countries’ death number and centers’ caseloads. We believe this survey does reflect current surgical practices, which highlights the emerging problems caused by COVID-19 patients.

In conclusion, this large international survey captured the global surgical practice under the COVID-19 pandemic and highlighted the insufficient preoperative screening of COVID-19 in the current surgical practice. We strongly believe that in the coming phase of pandemic, during which many medical centers will resume elective surgeries, a call for action in surgical departments is needed in global plans for infection control. More intensive screening programs will be necessary to prevent new potentially catastrophic outbreaks of infection in hospitals.
